# Rabies: Perils and Prevention, an Insight into Practices and Perception in Residents of Pakistan

**DOI:** 10.52547/ibj.3841

**Published:** 2022-12-19

**Authors:** Saif Ullah Shaikh, Faryal Zaidi, Zahida Shaikh, Muhammad Faisal Fahim

**Affiliations:** 1 Bahria University Health Sciences, Karachi, Pakistan;; 2 Liaquat University of Medical & Health Sciences, Jamshoro, Pakistan

**Keywords:** Inflammation, Interleukin-8, cytokines, Tumor necrosis factor-alpha

## Abstract

**Background:**

There is a sheer lack of knowledge in treating rabies in Pakistan. To decrease the number of victims every year, immunization and awareness programs are the basic necessities of Pakistani population. The aim of this study was to highlight the lack of learning strategies and how to overcome this problem, so as to eliminate rabies.

**Methods:**

This cross-sectional study was conducted on 692 respondents, aged 8-50 years, in Karachi city of Pakistan from January 2022 to June 2022. The study was based on demographic characteristics and basic knowledge of rabies, mode of transmission, clinical signs, and range of animal host species. Binary logistic regression analysis was performed to know the risk factor of rabies among different age groups, marital status, occupation, etc.

**Results:**

Results revealed that all the age groups were at risk of the wrong knowledge about rabies, odds = 1.182 and odds = 1.775 for 20-30 and 31-40 years of age, respectively; however, 31-40 years were at the high risk of showing odds=3.597 (95% C.I 1.621-7.983). The correlation of occupation was also checked with rabies knowledge. Only doctors (odds = 1.396) and students (odds = 1.955) showed their unawareness about rabies.

**Conclusion:**

This study highlights the grave situation that holds the country in the form of rabies. Through this study we aspire to raise awareness regarding the transmission, spread, and control of rabies.

## INTRODUCTION

Rabies is one of the leading animal-transmitted diseases in the subcontinent region; however, very little is known about this condition in general population^[^^1^^]^. Every year, rabies contributes to about 2,000 to 5,000 deaths in Pakistan alone^[^^2^^]^ and 55,000 at a global level^[^^3^^]^. The main agent of this disease is the rabies virus, which belongs to the *Lyssavirus* genus of the Rhabdoviridae family^[^^4^^]^. Rabies is caused primarily when a person is bitten by an infected dog^[^^3^^]^ and can affect all mammals^[^^5^^]^. The viral transmission is mainly through the saliva of an infected animal. Once bitten by the rabid animal, a person can present with flu-like symptoms and headache, sometimes progressing to hallucinations and paralysis as it mainly affects the brain and the spinal cord^[^^4^^]^. 

In South Asian countries, the number of unvaccinated dogs is on the rise, and unsatisfactory sanitation increases the incidence of rabies to a further degree^[^^6^^]^. Moreover, the rate of the population, particularly men and children, being bitten by a rabid dog has been observed to raise during summer^[^^7^^]^.

While rabies remains a definite problem in Pakistan, limited options are available for its control^[^^8^^]^. To date, the only method of addressing the rabies problem seems to be the killing stray dogs, as well as immunization program to the victims^[^^8^^]^. However, Pakistan is facing a vaccine shortage, and the number of infected victims keeps on increasing. Therefore, killing the rabid dogs appears to be the only easy and timely solution^[^^9^^]^. 

Due to the failure of disease controlling programs, rabies still remains an endemic disease in Pakistan^[^^5^^]^. Financial and political conflicts at the country level are another setback in dealing with rabies. There is also a significant negligence in the awareness of the disease due to which many cases are not dealt with timely medical intervention^[^^7^^]^. The basic treatment must include cleansing and irrigation of the wound, hemostasis, prophylactic antibiotics for high risk cases, and post exposure immunization^[^^10^^]^. However, since most doctors are practicing as general physicians, rather than specialists, they have a lack of knowledge about treating the dog bite appropriately^[^^10^^]^. Therefore, inadequate medical training, lack of awareness, and shortage of available vaccines contribute to the overall high mortality rate^[^^7^^]^. The aim of this study was to highlight the population lacking awareness of the disease and also bring to attention the possible recommendations to eradicate rabies.

## MATERIALS AND METHODS

This cross-sectional survey with non-probability purposive sampling was conducted at the health center Cantonment Board Clifton, South Circular Avenue DHA phase II, Karachi, Pakistan, from January 2022 to June 2022. The sample size was calculated by using online software openEpi (http://www.openepi.com/ Menu/OE_Menu.htm) with 95% CI and 5% margin of error. Hypothesized prevalence was considered as 50% to draw a large sample size, which was estimated to be 692. 

A Google doc online survey was conducted throughout all the provinces of Pakistan via social media platforms (Whatsapp, Facebook etc.). Approximately 1,000 forms were distributed online all over Pakistan to achieve sample size of 692 respondents. Subject consent forms were also distributed among the participants prior to the study. The samples included people of both genders, aged between 8 to 50 years, having different educational and professional backgrounds and residing in both rural and urban regions of four provinces (Sindh, Punjab, Baluchistan and Khyber Pakhtunkhwa) of Pakistan. These study regions were selected because of their differences in topography and daily interaction with animals, which both contribute to rabies spread. The young (less than 18 years of age) were surveyed as a focus group because of their highest exposure rate to rabies disease. Participants with incomplete submitted records and those who did not give any consent were excluded from the study. 

The questionnaire used in this study included two sections. Section-I was based on the collection of data using self-structured questionnaire about demographic characteristics and basic knowledge of rabies. The section-II included questions designed to acquire each respondent’s knowledge and awareness regarding rabies disease, mode of transmission, clinical signs, and range of animal host species. It was a self-administrated questionnaire with open and closed ended questions.

Statistical analysis was carried out using statistical package for Social Sciences (SPSS) version 25.0. Qualitative variables were presented as frequency and percentages. Questions about knowledge and perception of rabies were compared with occupation, and percentages were observed. Validity and reliability of the data checked through Cronbach’s alpha test as found to be 0.8 (good level). To observe the significance between all demographic variables (age, marital status, occupation, and province of residence) and the knowledge of rabies, we performed using binary logistic regression analysis. *p* value ≤0.05 was considered as statistically significant. 

## RESULTS

A total of 692 questionnaires with full responses were received from different provinces of Pakistan. There were 160 (20%) responses from Baluchistan, 150 (18.7%) from Khyber Pakhtunkhwa, 244 (30.4%) from Punjab, and 248 (30.9%) from Sindh. 

Based on Table 1, all the age groups, including 20-30 (odds = 1.182), 31-40 (odds = 1.775), and 41-50 (odds = 3.597) years were at risk of wrong knowledge about rabies (95% CI: 1.621-7.983), indicating that people in aged 31-40 years are at high risk of having rabies because of their insufficient knowledge of rabies. No risk differences were observed regarding the knowledge of rabies on marital status. Comparison between occupation and rabies knowledge showed that only doctors (odds = 1.396) and students (odds = 1.955) had insufficient knowledge of rabies and were at moderate risks. Residents of different provinces were also compared with rabies knowledge. Punjab was found to have high risk of being infected by rabies because of unawareness regarding rabies (odds = 9.247; 95% CI: 1.325-18.362). 

As represented in Table 2, in response to the question “what is rabies?”, 93.9% responded that it is a disease, while 6.1% considered it as a change in behavior. 

When asked about whether rabies is transmitted from one human to another, 35.1% responded yes, while 44.2% responded no, and 20.7% were not sure about this question. When asked about whether rabies is a deadly disease, 76.3% agreed, 8.1% disagreed, while 15.6% were not aware about the question. In response to the question “which organ(s) of the body is/are infected by rabies”, 3.8% reported reproductive system, 3.9% gastrointestinal tract, and 8.5% cardiovascular system, while the majority (83.8%) confirmed that the central nervous system is mostly infected with rabies. When asked about how rabies is transmitted to humans, the response of 6.8% was via mucus membranes in the eyes, 20.2% through living with rabid animal, 14.6% by eating rabid animal-like meat, and 58.4% via broken skin. Regarding the question “what happens if rabies was not treated in humans?”, 79.2% replied that person dies, whereas 20.8% were unsure/unaware about the question. When asked about the prevention of rabies, 77% of respondents agreed that it is preventable by vaccination of dogs, 11.4% stated that it cannot be prevented, and 10.8% were unsure. Details of the questions are illustrated in Table 2.

**Table 1:** Risk of rabies knowledge compared to demographic characteristics

**Demographics vs. ** **knowledge about rabies**	**B**	**S.E.**	**Wald**	***p*** ** value**	**Exp (B)**	**95% CI for EXP(B)**	
						**Lower**	**Upper**
**Age**							
<20	0.168	0.627	0.072	0.789	1.182	0.346	4.038
20-30	0.574	0.823	0.486	0.486	1.775	0.354	8.899
31-40	1.280	0.407	9.907	0.002	3.597	1.621	7.983
41-50					1		
	
**Marital status**							
Single	-0.530	0.623	0.722	0.395	0.589	0.173	1.998
Married					1		
	
**Occupation**							
Doctor	0.333	0.703	0.225	0.635	1.396	0.352	5.537
Government employee	-2.366	1.017	5.415	0.020	0.940	0.013	0.689
Housewife	-2.457	1.296	3.593	0.058	0.860	0.007	1.087
Private job	-0.795	0.906	0.769	0.380	0.452	0.076	2.668
Student	-0.047	1.092	0.002	0.966	1.955	0.112	8.123
Teacher					1		
	
**Province of residence**							
Baluchistan	0.322	0.499	0.417	0.519	1.380	0.519	3.669
Khyber Pakhtunkhwa	0.165	0.476	0.121	0.728	1.180	0.464	2.999
Punjab	2.224	1.255	3.141	0.076	9.247	1.3256	18.362
Sindh					1		

^*^Binary logistic regression analysis applied to see the significance at *p* ≤ 0.05. B, beta; S.E., standard error; Sig., Exp (B), odd ratios

## DISCUSSION

Our findings demonstrated that the young population (aged 31-40 years) was mostly at high risk[^11^^,^^12^^]^ of having rabies. A similar study carried out in 2020 by Ahmed and colleagues^[^^3^^]^ on 1466 individuals living in different provinces of Pakistan also revealed that the young population (aged 18 years) was mostly affected by rabies, when compared to adults. Likewise, in Asia and Africa alone, about 14% of the victims of rabies were under 15 years of age^[^^11^^,^^13^^]^.

Demographically, rabies is most commonly found in Punjab more than other provinces of Pakistan owing to the dense population. One reason for the association of geography with the incidence of disease could be the fact that rural areas^[^^12^^]^ have limited access to vaccination and general education about rabies^[^^3^^,^^14^^]^. The results also showed that the majority of the population was aware that rabies is a disease rather than a change in behavior. As for rabies transmission, most of the doctors and housewives disagreed on transmission route (from one person to another), while government employees agreed; however, teachers were unsure. Regarding the influence of rabies on body systems, most doctors, housewives, and teachers confirmed the transmission via broken skin. A significant number of teachers and housewives was also thought that living with a rabid animal contributes majorly to the transmission. Overall, these results strongly suggest a great deal of ambiguity in the awareness about the disease^[^^15^^]^.

The present study highlights the importance of immunization and wound management. If prompt treatment is not given, and the wound is not washed immediately, the affected person has a high chance for death^[^^2^^]^. While the popular opinion was that the affected person will die if not treated, there were still a great number of people who did not have any knowledge about the disease. Most of the population agreed on the prevention of rabies by vaccination of dogs^[^^11^^]^, but housewives disagreed or were skeptical.

**Table 2:** Knowledge of rabies in context to occupation

**Questions asked from respondents**	**Responses (%)**		**Occupation**					
		**Doctor(n = 191)**	**Government employee ** **(n = 10)**	**Housewife(n = 9)**	**Private job(n = 14)**	**Student(n = 429)**	**Teacher(n = 39)**	**Total (n = 692)**
What is Rabies?	Change in behavior	6.3	50.0	22.2	28.6	3.5	10.3	6.1
	Disease	93.7	50.0	77.8	71.4	96.5	89.7	93.9
								
Is rabies transmitted from one human to another?	No	49.7	20.0	66.7	35.7	41.5	51.3	44.2
	Not sure	23.0	30.0	0.0	28.6	21.0	5.1	20.7
	Yes	27.2	50.0	33.3	35.7	37.5	43.6	35.1
								
Is rabies a deadly disease?	No	8.4	0.0	22.2	0.0	8.9	0.0	8.1
	Not sure	2.6	0.0	0.0	14.3	23.1	5.1	15.6
	Yes	89.0	100.0	77.8	85.7	68.1	94.9	76.3
								
Which system of the body is infected by rabies?	CNS	95.3	100.0	77.8	71.4	81.4	56.4	83.8
	CVS	3.1	0.0	22.2	0.0	10.3	17.9	8.5
	GIT	1.6	0.0	0.0	14.3	2.8	25.6	3.9
	Reproductive system	0.0	0.0	0.0	14.3	5.6	0.0	3.8
								
How is rabies transmitted to humans?	Broken skin	73.8	50.0	66.7	50.0	51.5	61.5	58.4
	Eating rabid animal-like meat	5.8	0.0	0.0	21.4	16.8	38.5	14.6
	Living with rabid animal	14.1	20.0	33.3	14.3	24.7	0.0	20.2
	Mucus membranes in the eyes	6.3	30.0	0.0	14.3	7.0	0.0	6.8
								
What happens if rabies is not treated in humans?	Not sure/don't know	24.6	0.0	22.2	42.9	20.3	5.1	20.8
	Person dies	75.4	100.0	77.8	57.1	79.7	94.9	79.2
								
Can Rabies be prevented by vaccination of dogs?	No	2.1	0.0	44.4	0.0	15.6	10.3	11.4
	Not sure	5.2	0.0	33.3	0.0	14.0	5.1	10.8
	Yes	92.7	100.0	22.2	100.0	70.4	84.6	77.7

CNS, central nervous system; CVS, cardiovascular system; GIT, gastrointestinal system

In addition, a fair number of private job employees were uncertain about the use of antivirals and vaccination. According to this observation, it could be deduced that most people agree on immunization. However, there was a massive disagreement in this matter^[^^16^^]^, which has to be addressed to completely eliminate the disease.

Timely management of a wound is one of the crucial steps in the prevention of rabies^[^^3^^]^; however, in Karachi, the biggest city of Pakistan, proper wound management is not often carried out^[^^17^^]^. While most doctors and government employees agreed on postexposure vaccine, a great number of private job employees and students were unsure, which further highlights the inadequacy of knowledge regarding the initial treatment^[^^11^^,^^12^^,^^14^^]^. Another major setback, especially in the rural areas, is referring to a spiritual healer rather than a doctor^[^^18^^]^. This matter has also been shown by the results that a great percentage of population, particularly the government employees, considered a spiritual healer as the primary care provider in rabies cases. A similar behavior was also observed in a different part of the world. In Nigeria, as highlighted by Rine^[^^11^^]^, more than half of the study participants would consider traditional modes of treatment rather than proper medical intervention.

When taking first aid into consideration, which should be known to almost everyone living with animals[^19^^]^, not much of the population was aware about it. While the majority believes washing with water and soap^[^^11^^,^^17^^]^ is the initial wound treatment, most government employees consider tying with cloth as the ideal first aid. Similarly, most of the population agreed on a solution for providing a proper service for rabies control, but government employees disagreed. Reasons for this disagreement could be due to political unrest^[^^20^^]^ and pressures from the governing parties. Therefore, these areas need to abide by strict rules and regulations introduced by the governing bodies, in order to completely eradicate rabies.

The current study highlights the grave situation that holds the country faces in the form of rabies. While most countries of the world have combated the disease by the aid of prompt immunization program, Pakistan still struggles with this condition. There could be multiple reasons for this issue; one of the main reasons is the sheer lack of awareness. The goal is to eradicate rabies from the face of the earth by increasing awareness, and taking necessary precautionary measures and, thus, relieve the sufferings of mankind on a global level. 

## DECLARATIONS

### Acknowledgments

We acknowledge the Cantonment Board Clifton Healthcare Center for their cooperation in provision of data and ethical statement.

### Ethical statement

The study protocol was approved by the Ethics Committee of Cantonment board Clifton, health center DHA, Phase II, Karachi, Pakistan (ethical code: CBC/HC/No.0258). Consent forms were reeived from all participants. 

### Data availability

Our data is confidential, and we cannot share raw data; however, we can provide SPSS output sheet for verification of the results.

### Author contributions

SUS: principal investigator, drafting the manuscript, and critical revision of the manuscript, intellectual content; FZ: study design, drafting the article and rephrasing; ZS: data collection; MFF: compilation of data and results.

### Conflict of interest

None declared.

### Funding/support

This study has received no financial support from any institute or organization.
